# Paget’s Disease of the Bone and Lynch Syndrome: An Exceptional Finding

**DOI:** 10.3390/diagnostics13122101

**Published:** 2023-06-17

**Authors:** Ana-Maria Gheorghe, Laura-Semonia Stanescu, Eugenia Petrova, Mara Carsote, Claudiu Nistor, Adina Ghemigian

**Affiliations:** 1 C.I. Parhon National Institute of Endocrinology, 020021 Bucharest, Romania; anamaria.gheorghe96@yahoo.com (A.-M.G.); stanescu_laurasemonia@yahoo.com (L.-S.S.); jekined@yahoo.com (E.P.); adinaghemi@yahoo.com (A.G.); 2 PhD Doctoral School, Carol Davila University of Medicine and Pharmacy, 050474 Bucharest, Romania; 3 Department of Endocrinology, Carol Davila University of Medicine and Pharmacy, 050474 Bucharest, Romania; 4 Department 4—Cardio-Thoracic Pathology, Thoracic Surgery II Discipline, Carol Davila University of Medicine and Pharmacy & Thoracic Surgery Department, Dr. Carol Davila Central Emergency University Military Hospital, 050474 Bucharest, Romania

**Keywords:** Paget’s disease of the bone, Lynch syndrome, diagnosis, endocrine, zoledronate, *MMR* gene, surgery, *SQSTM1* gene, whole exome sequencing, bone turnover marker

## Abstract

Our objective is to present an exceptional case of a patient diagnosed with Paget’s disease of the bone (PDB) while being confirmed with Lynch syndrome (LS). A 44-year-old woman was admitted for progressive pain in the left forearm 2 years ago, and was partially relieved since admission by non-steroidal anti-inflammatory drugs. Suggestive imaging findings and increased blood bone turnover markers helped the diagnosis of PDB. She was offered zoledronate 5 mg. She had two more episodes of relapse, and a decision of new medication was taken within the following years (a second dose of zoledronate, as well as denosumab 60 mg). Her family history showed PDB (mother) and colorectal cancer (father). Whole exome sequencing was performed according to the manufacturer’s standard procedure (Ion AmpliSeq™ Exome RDY S5 Kit). A heterozygous pathogenic variant in the *SQSTM1* gene (c.1175C>T, p.Pro392Leu) was confirmed, consistent with the diagnosis of PDB. Additionally, a heterozygous pathogenic variant of *MSH2* gene (c.2634+1G>T) was associated with LS. The patient’s first-degree relatives (her brother, one of her two sisters, and her only daughter) underwent specific genetic screening and found negative results, except for her daughter, who tested positive for both pathogenic variants while being clinically asymptomatic. The phenotype influence of either mutation is still an open issue. To our current knowledge, no similar case has been published before. Both genetic defects that led to the two conditions appeared highly transmissible in the patient’s family. The patient might have an increased risk of osteosarcoma and chondrosarcoma, both due to PDB and LS, and a review of the literature was introduced in this particular matter. The phenotypic expression of the daughter remains uncertain and is yet to be a lifelong follow-up as the second patient harbouring this unique combination of gene anomalies.

## 1. Introduction

### 1.1. Paget’s Disease of Bone

Paget’s disease of the bone (PDB), a slowly progressive, chronic disorder, is characterized by impaired bone remodelling in one (monostotic) or more skeletal sites (polyostotic) [1]. It was first described in 1876 by Sir James Paget as “osteitis deformans” based on the evaluation of several cases, which had in common curved and misshaped weight-bearing bones and a thickening of the skull [2]. PDB is the second most frequent metabolic disease of bone (after osteoporosis), affecting about 1–8% of the general population; it is more prevalent in males than females, with a steep increase in prevalence after 50 years of age in both sexes (it has been reported almost worldwide, with large differences in prevalence) [3]. The highest rates have been found in people of British descent, particularly in England, Western Europe, Australia, New Zealand, and North America [4]. This prevalence pattern was established by examining the skeletal remains found in the archaeological population [5]. Recent evidence suggests a decrease in prevalence and severity [3,6].

The cause of these events is not completely understood. Theories involve demographic and sociological changes, as well as environmental triggers, genetic and epigenetic factors [7]. Both genetic causes and environmental contributors are involved. The most important susceptibility gene for PDB is *Sequestosome* 1 gene (*SQSTM1*), detected in up to 40% of familial cases and about 10% of sporadic cases. Its role in bone metabolism is well known, by encoding p62 protein, involved in activating the nuclear factor kappa B (NF-kB) signalling pathway, with a role in osteoclastogenesis. Around 30 different germline pathogenic variant of the *SQSTM1* have been described. There is an autosomal dominant pattern of inheritance with incomplete penetrance. *SQSTM1* pathogenic variants are generally heterozygous and are associated with more severe forms of the disease [1,8,9]. Other pathogenic variants involve *ZNF687*, *PFN1*, *TNERSF11A*, or *FKBP5* genes. Recent Genome Wide Association Studies (GWAS) identified susceptibility genes, loci and single nucleotide variants, including rs1561570 within the *OPTN* gene, rs484959 near the *CSF1* gene, rs3018362 near the *TNFRSF11A* gene, rs5742915 within the *PML* gene, and others [1]. Another role in disease development may be played by epigenetic modifications of genes involved in bone metabolism such as RANKL (receptor activator of nuclear factor-kB ligand), OPG (osteoprotegerin), HDAC2, DNMT1, and SQSTM1 [10]. Among the environmental factors, we mention exposure to zoonoses (canine distemper) and viral infections (measles and respiratory syncytial virus), environmental toxins, vitamin D deficiency in childhood and skeletal injury, and repetitive mechanical loading [11,12,13,14,15].

Osteoclasts are the main cells affected in PDB. In Paget lesions, osteoclasts are increased both in number and size. Their precursors are hypersensitive to osteoclastogenic factors such as 1,25 dihydroxy-vitamin D, RANKL, and tumour necrosis factor-α [16]. These cells are highly metabolically active. In response to the rapid bone resorption, bone formation is also accelerated. The increased bone turnover due to increased osteoclastic activity and disorganised bone formation by osteoblasts is reflected by the lamellar and woven microarchitecture of the bone. As a consequence, various complications may occur, including bone pain, deformity, secondary osteoarthritis, and pathological fractures [17,18]. Bones are less mineralised, thus having less stiffness [19,20], but others reported them as being more mineralised [21].

Symptoms and signs are present in about 75% of patients [22,23]. Mostly, they include: bone pain in the affected region; possible warmth and redness accompanying pain due to an increased vascularisation; bone enlargement and deformities such as bowing of the legs, skull enlargement, and kyphosis may be present; and the axial skeleton, which is most commonly affected. Other clinical elements are: constipation, abdominal pain, and hearing loss, as well as headache, especially related to skull involvement [10,24,25].

Biochemical tests reveal increased bone turnover. Increased bone formation is reflected by high levels of serum alkaline phosphatase (ALP), produced by osteoblasts, bone-specific ALP, procollagen type 1 amino-terminal peptide (P1NP), and osteocalcin. Bone resorption is reflected by elevated urinary N-telopeptide (NTx) and type I collagen C-telopeptide (CTX) [26,27]. Disease activity may be best monitored by P1NP levels; in case it is unavailable, total ALP is a useful substitute [26]. Moreover, ALP can be used for PDB screening [17].

Imaging techniques used in diagnosing PDB include plain X-rays and bone scintigraphy. X-rays of the affected sites may show lytic lesions, especially in the early stages of the disease. Suggestive lesions are: cortical thickening, trabecular thickening, and a loss of distinction between cortex and medulla, osteosclerosis, bone expansion, and bone deformities. Bone scintigraphy is used to evaluate disease extent and may show intense tracer uptake. A negative result does not exclude PBD [8,17,18].

The increased resorption leads to a release of calcium from the bones and poses the threat of hypercalcemia, especially in immobilised patients with multiostotic disease [28]. Differential diagnosis of hypercalcemia includes primary hyperparathyroidism, which rarely co-exists with PDB or malignant (paraneoplastic) syndromes accompanying different cancers. Secondary hyperparathyroidism due to anti-resorptive treatment, vitamin D deficiency or increased bone formation may also occur. Elevated bone formation leads to a high calcium uptake in skeleton, which stimulates the secretion of PTH (parathyroid hormone). Increased PTH, especially in hyper-vascular forms, stimulates osteoclasts, providing calcium for osteoblasts; thus, secondary hyperparathyroidism with normo- or hypercalcemia may be identified [29]. Vitamin D deficiency in PDB may be a result of increased mineralisation due to osteoblastic activity (as part of the bone formation phase) [30]. Vitamin D receptors of osteoclasts precursors in PDB seem to have a higher sensitivity to 1,25-hydroxy-vitamin D [31].

Treatment aims to restore normal bone remodelling, relieve pain, and prevent fractures and other complications. Traditional drugs such as bisphosphonates and, most commonly, a single infusion with zoledronate are used [32]. Bisphosphonates inhibit farnesyl pyrophosphate synthase, an enzyme involved in cytoskeleton synthesis, leading to osteoclasts apoptosis (most potent enzyme inhibitor being zoledronate) [33,34]. Non-isomerised (alpha)/isomerised (beta)-CTX ratio is increased both in bone and urine, with alpha-CTX having a greater distribution within woven bone, thus leading to turnover inhibition [35,36]. The urinary ratio of alpha/beta-CTX was shown to normalise under zoledronate, also suggesting an improvement of bone microarchitecture [37]. Most frequently, ALP is assessed, following the response to therapy, in addition to symptom-based management, which is equally important [36,38]. Zoledronate poses the risk of hypocalcaemia/secondary hyperparathyroidism, especially in patients with high disease activity due to its retention in areas with an increased bone turnover [39].

### 1.2. Lynch Syndrome

Lynch syndrome (LS) (hereditary nonpolyposis colorectal cancer) represents the most common inherited cancer susceptibility syndrome. It is inherited in an autosomal dominant pattern and is associated with colorectal cancer, accounting for 3–5% of new diagnoses [40,41]. LS is also associated with extra-colonic cancers, especially endometrial, but also gastric, hepatobiliary, pancreatic, ovarian, etc. [41,42]. Underlying germline mutations are DNA mismatch repair *(MMR) genes* (*MLH1*, *MSH2*, *MSH6*, *PMS2*, and *EpCAM*); their pathogenic variants prevent the proper repair of DNA errors during replication; as the abnormal cells continue to divide, the accumulation errors can lead to uncontrolled cell growth and possibly cancer [43,44]. The diagnosis of LS involves MMR gene testing as well as tumour tissue analysis for the identification of MMR proteins (and/or microsatellite instability) [42,45,46].

### 1.3. Aim

Our objective is to present an exceptional case of a patient confirmed with PBD while being confirmed with LS (each disease associated to a specific pathogenic variant, one from the mother and the other from the father). To our current knowledge, no similar case has been published before. Other mostly interesting aspects involve the presence of a single bone PBD type, yet, with a relapsing evolution that required additional prompt medical intervention, and the co-presence of metabolic concerns in relationship with PBD.

## 2. Case Report

### 2.1. Presentation: On Admission

**Figure 1:**
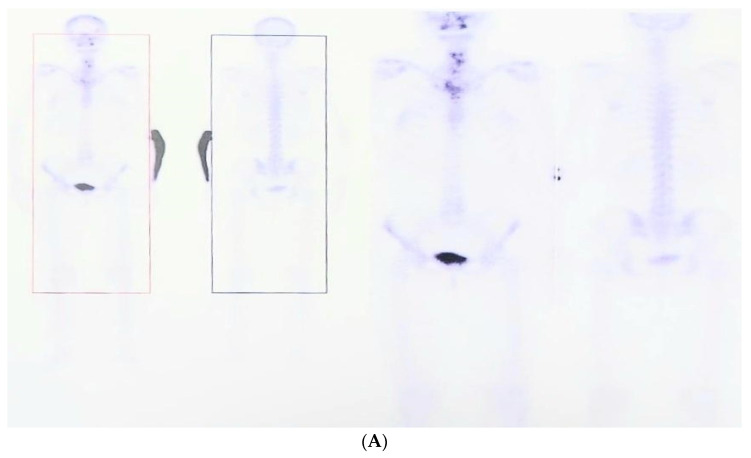

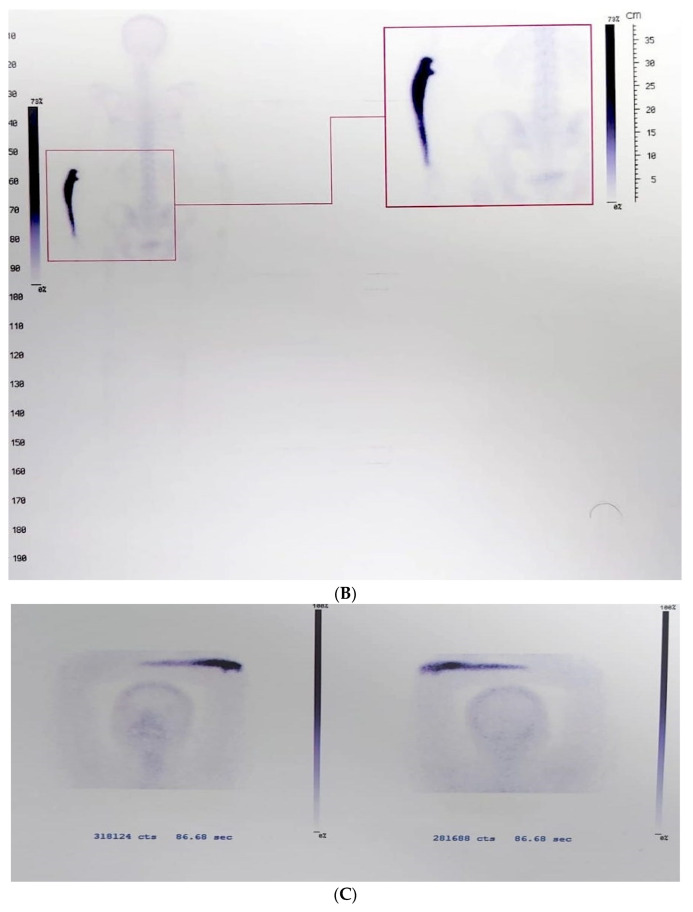

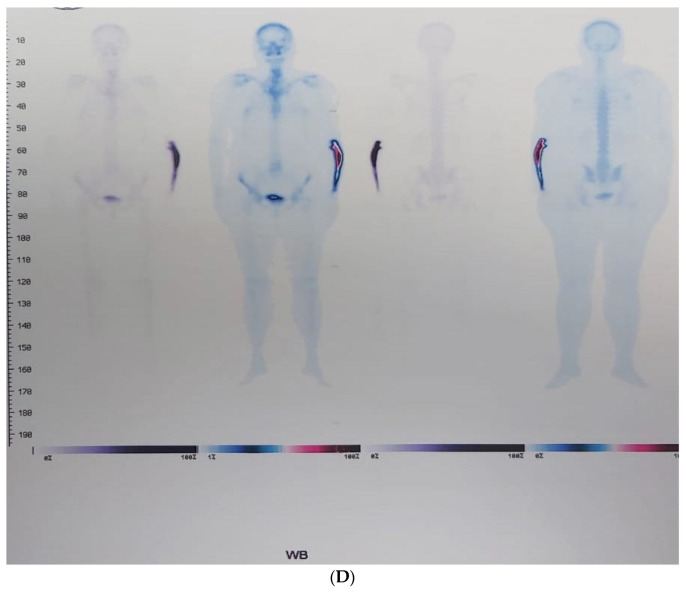
Technetium-99m methylene diphosphonate bone scan showing increased uptake in the left forearm (**A**–**D**), images captured at different times amid tracer uptake). (**A**) Whole body capture. (**B**) Forearm capture. (**C**) Head capture. (**D**) Left forearm capture.

A 44-year-old woman was admitted in November 2019 due to suspected PDB. The patient presented with progressive pain in the left forearm occurring during the past 2 years, and was relieved by non-steroidal anti-inflammatory drugs. The pain was accompanied by swelling of the same site. Prior to admission, the patient underwent laboratory tests, which showed a high serum total ALP of 392 U/L (normal: 35–104 U/L) and a technetium-99 m methylene diphosphonate (99mTc-MDP) bone scan, which revealed increased uptake in the affected left forearm (Figure 1).

The patient’s medical history included well-differentiated rectal adenocarcinoma diagnosed at the age of 34 years, treated with chemotherapy and radiotherapy, leading to secondary amenorrhea, and an endoscopically removed oesophageal papilloma. In addition, she had three lumbar herniated discs, for which she underwent surgery, and suffered from autoimmune chronic thyroiditis with normal thyroid function.

Regarding her family medical history, the patient reported that her mother was diagnosed with polyostotic PDB, which affected her skull, femur, and spine, at the age of 44 years. Furthermore, her father died from colorectal cancer at 47 years of age. No specific genetic testing was performed so far.

Currently, her physical examination revealed a swelling of the proximal extremity of the left forearm and tenderness to palpation. Biochemical workup showed vitamin D deficiency, a slightly elevated serum calcium of 10.3 mg/dL (normal: 8.4–10.2 mg/dL), and high 24 h urinary calcium of 0.33 g/24h (normal: 0.07–0.3 g/24-h). Serum phosphorus and PTH were within normal range, so were the kidney and liver functions. Bone turnover markers were elevated: total ALP of 347 U/L (normal: 40–150 U/L), P1NP of 203.4 ng/mL (normal: 20.25–76.31 ng/mL), and ßCTx of 0.67 ng/mL (normal: 0.162–0.436 ng/mL) (Table 1).

**Figure 2:**
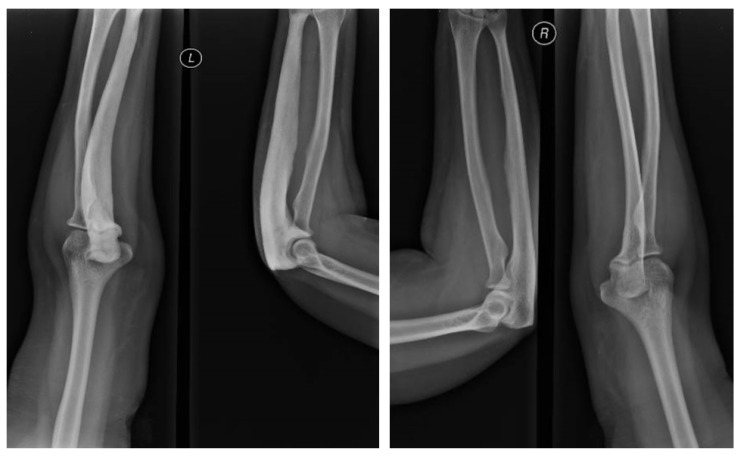
X-ray examination: Enlargement of the proximal left ulna and cortical thickening, suggestive of PBD (X-ray of the left (**L**) and right (**R**) forearm).

Upper-limb X-rays confirmed the enlargement of the proximal region of the left ulna with increased density and cortical thickening. There were no signs of fractures (Figure 2).

Central dual-energy X-ray absorptiometry (DXA) confirmed osteopenia (Table 2).

**Figure 3:**
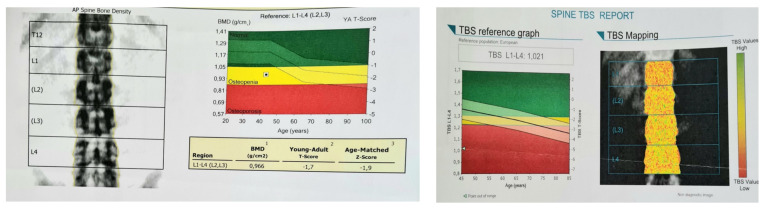
DXA assessment; (**left**) lumbar spine scan showing osteopenia according to *t*-score; (**right**) TBS report confirming a degraded microarchitecture according to low TBS of 1.021.

DXA-based trabecular bone score (TBS) was low (at 1.021) (Figure 3).

### 2.2. Gene Testing: Confirmation of Harbouring Two Pathogenic Variants

**Figure 4:**
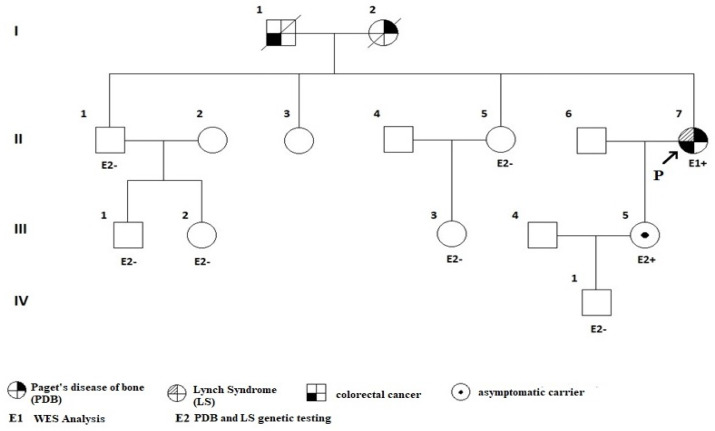
Family tree of the proband: a 44-year-old female confirmed (II.7.) with PDB and LS (patient number 7), as well as her daughter (patient number III.5); she inherited PDB from her father and LS from her mother. Abbreviations: P = proband (index-case); PDB = Paget’s disease of the bone; LS = Lynch syndrome; WES = whole exome sequencing.

Following the genetic counselling, whole exome sequencing (WES) was performed according to the manufacturer’s standard procedure (Ion AmpliSeq™ Exome RDY S5 Kit, Bucharest, Romania). A heterozygous pathogenic variant in the *SQSTM1* gene (c.1175C>T, p.Pro392Leu) was confirmed, consistent with the diagnosis of PDB [47,48,49,50]. Additionally, a heterozygous pathogenic variant of *MSH2* gene (c.2634+1G>T) was associated with LS [51,52]. The patient’s first-degree relatives (her brother, one of her sisters, and her daughter) underwent specific genetic screening. Only her daughter was tested positive for both pathogenic variants while being clinically asymptomatic. One of her sister’s assessments were not available. Both of her parents died of medical conditions (her father was most probably carrying the pathogenic variant for LS, while her mother of PBD) (Figure 4).

### 2.3. Case Management

**Figure 5:**
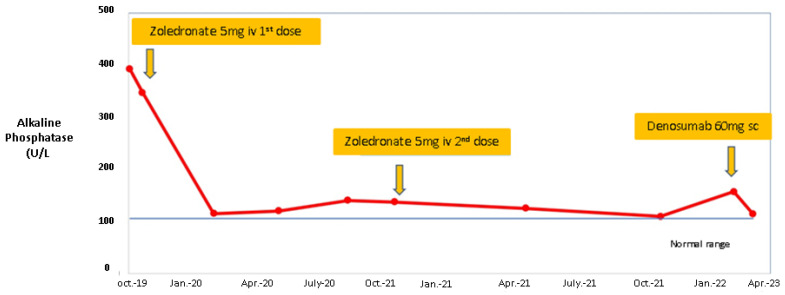
The trend line of ALP levels during treatment, starting with the first administration of zoledronic acid in a 44-year-old female with PDB and LS, followed by a second 5 mg dose and then by initiation of denosumab.

For PDB, a single 5 mg dose of zoledronate was administered in addition to adequate vitamin D supplementation. At three-month follow-up, the patient showed clinical improvement, and a significant decrease in bone turnover markers such as ALP to 115 U/L (normal: 40–150 U/L), P1NP of 75.99 ng/mL (normal: 20.25–76.31 ng/mL), and ßCTX of 0.29 ng/mL (normal: 0.162–0.436 ng/mL). At one-year follow-up, a mild elevation of ALP to 160 U/L (normal: 40–150 U/L), P1NP of 81.88 ng/mL (normal: 20.25–76.31 ng/mL), and ßCTX of 1.01 ng/mL (normal: 0.162–0.436 ng/mL) were noted; thus, a second intravenous infusion of 5 mg zoledronate was offered to the patient. At eighteen-month follow-up, after the second dose of zoledronate, the patient relapsed and presented with a recurrence of bone pain. A decision of switching to subcutaneous injection of denosumab 60 mg was made (Figure 5).

For the management of LS, we recommend to follow the general multidisciplinary protocol, including annual colonoscopy and esophagogastroduodenoscopy, and screening for endometrial and ovarian cancer by endometrial sampling and transvaginal ultrasound. Similar periodic check-up is required for the family members harbouring the pathogenic variants. Lifelong surveillance is mandatory. 

## 3. Discussion

This case, considered unique for its kind, connects several multidisciplinary chapters that are worth to be mentioned as follows:

### 3.1. The First Reported Case of PDB (Maternal Inheritance) and LS (Paternal Inheritance)

To our current knowledge, this is the first case of PDB and LS co-existing in one patient. We searched three databases (PubMed, Google Scholar, and Web of Science) using the keywords: “Paget” (alternatively, “Paget bone disease”) in association with “Lynch”, (alternatively, “Lynch syndrome”). Even though PDB most commonly affects the axial skeleton [24], our patient presented with monostotic disease affecting her forearm—this being among the most commonly affected sites. One retrospective study conducted on 69 patients with PBD revealed that the upper limb was affected in a relatively small percentage of patients (5.8%) [53]. Clinical manifestations are in contrast with her mother, who showed polyostotic disease involving the skull, femur, and spine. One similarity between them was the age at diagnosis, both being diagnosed around the age of 44. Long-term follow-up will reveal whether the forearm remains the only affected site or a polyostotic form will be identified.

### 3.2. Genetic Considerations

The patient was diagnosed with two different pathogenic variants, one heterozygous pathogenic variant in the *SQSTM1* gene (c.1175C>T, p.Pro392Leu), associated with PDB [47,48,49,50], and a heterozygous germline mutation in the *MSH2* gene (c.2634+1G>T), associated with LS [51,52]. *P392L* is the most common pathogenic variant linked to PDB [47,48]. The involvement of *P329L* in PDB development also includes osteoclasts activation and differentiation [48,49]. Even though it was linked to an increased osteoclastogenic potential, it did not seem sufficient to cause PDB, suggesting that other factors may play a role in disease development [47]. In addition, this gene is associated with disease severity and complications [50].

The co-occurrence of PDB and LS has not been described before. However, other cases of two different autosomal dominant diseases co-occurring, although similarly rare, have been described before [54,55]. The fact that both pathogenic variants were transmitted to the patient’s daughter raises the question whether it was utter chance or due to inter-chromosomal interactions (non-homologous chromosomal contacts), or other unknown factors may have played a role. It also remains uncertain how the developing phenotype may be influenced by inheriting both pathogenic variants. So far, it is difficult to predict how inheriting both pathogenic genes will affect our patient’s daughter, including the development of LS-associated manifestations, and how they will find their way to express at phenotype level.

### 3.3. Bone Turnover Markers’ Profile: From PDB Diagnosis to Outcome

Serum total ALP is not only recommended for screening for PDB, and predicting disease extent, but also for monitoring response to treatment, especially bisphosphonates [17]. ALP normalisation usually takes at least one month. The fastest response concerns bone resorption markers [56]. In our patient’s case, at the three-month check-up after the first dose of zoledronate, the level of total ALP value normalised, and likewise the other bone turnover markers: P1NP and ßCTx. In terms of long-term outcome, it appears that there are no significant differences between aiming at normalizing ALP and symptom-based management [36]. Despite the fact that we expected to achieve a durable response to bisphosphonates therapy, given the rapid decrease in blood bone turnover markers and a single bone-derivate PDB, the serum bone turnover markers increased after 1 year; thus, a second dose of zoledronic acid was considered. Of note, no further skeleton site was involved so far. Even if the current guidelines [17,28] recommend a single (5 mg) dose of zoledronate and there are no specific data regarding re-treatment, we prescribed an additional dose. The considerable interpatient variability of treatment response is probably due to a heterogeneous sensitivity of bone cells to therapy. After another year and a half, the disease relapsed. Because the bone turnover markers were above the reference range and the pain was not controlled with symptomatic medication, we recommend a dose of 60 mg of denosumab subcutaneously.

### 3.4. Denosumab Use in PBD

Denosumab prevents osteoclasts differentiation and survival by preventing the binding of RANKL to its receptor [57]. The OPG/RANK/RANK ligand system is usually not involved in PDB development according to current knowledge [58]. However, RANKL was shown to be expressed slightly higher in PDB affected sites [59]. Evidence regarding the use of denosumab for PDB is scarce [60,61,62,63,64,65]. We performed a review of the medical literature in mentioned databases (from inception to 2023), and identified six case reports, starting with 2012 [62] until 2018, the most recent [61], in which denosumab was used for PDB treatment (Table 3).

According to the literature research, most candidates of denosumab had a polyostotic disease [60,61,62,63,64]. In three cases, denosumab was prescribed because the subjects had a contraindication for bisphosphonates treatment due to renal function impairment [60,61,62]. Two patients received denosumab because of the presence of giant cell tumours (GCT) [63,64], and another patient due to developing adverse reactions to oral alendronate [65]. Most individuals received a dose of 60 mg denosumab [60,61,62,65], while one patient [64], who was suffering from GCT, received a higher dose of 120 mg. 

Due to poor adherence to calcium supplementation recommendations, one patient developed severe hypocalcaemia within 4 weeks following denosumab 60 mg [60].

In terms of PDB outcome, these patients displayed clinical and biochemical improvement, including ALP levels normalisation [60,61,62,65]. In one case [65], however, ALP levels rose again and further doses were needed. Bone scintigraphy showed decreased disease activity compared to baseline [62,65]. Overall, symptoms improved in all patients [60,61,62,63,64,65].

### 3.5. Cancer Risk in Patients with PDB: Focus on Osteosarcoma

Although a rare complication affecting about 1–1.4% of patients, osteosarcoma is linked to PDB, especially in patients with polyostotic disease and long-established disease [17,53,66,67]. Even though the molecular links between PDB and osteosarcoma are not yet entirely clear, the alterations in bone microarchitecture may contribute to the development and progression of osteosarcoma. Osteolytic activity increases porosity, while osteoblastic activity generates stiffness. Collagen fibres become disorganised and pores lose their regular alignment. These alterations of bone microarchitecture create “cancer niches”, which may favour the development of osteosarcoma as well as bone metastases from secondary sites [68]. Data concerning the risk of metastases from secondary sites in patients with PDB are scarce, and further research is needed.

Chondrosarcoma is another uncommon occurrence that has been reported in patients affected by PDB. Ferreira et al. [69] presented the case of an 84-year-old woman with pelvic chondrosarcoma as a first manifestation of monostotic PDB [69].

### 3.6. MMR Pathogenic Variants and Skeleton Findings: How Far to an Osteosarcoma?

While osteosarcoma is more common in PDB, neither type (osteosarcoma or chondrosarcoma) is traditionally associated with LS [70,71,72,73,74,75]. However, some studies suggested that LS might increase the risk of developing sarcomas, including osteosarcoma [71,72,73,74,75]. Moreover, *MSH* genes, especially *MSH2*, were previously linked to osteosarcomas, but to a low level of statistical evidence [70] (Table 4).

Overall, we identified five such papers (case series and reports) introducing data on skeleton malignancies in subjects diagnosed with LS [71,72,73,74,75]. Of course, the risk of osteosarcoma seems a distant scenario to connect LS to PDB since a direct link has not been established yet. In our case, the presence of the two pathogenic variants seems related to the inheritance from each patient. Due to the novelty of the situation, it is difficult to predict if one pathogenic variant will influence the clinical presentation of the other as well as anticipating the phenotype in the young daughter’s case. Additionally, whether a higher risk of osteosarcoma is connected when displaying both pathogenic variants remains uncertain. 

One prospective observational study investigating cancer risk among carriers of LS-associated pathogenic variants identified 12 cases of osteosarcomas (seven females and five males), among 1808 tumours in 6350 path-MMR carriers. The mean age in osteosarcoma-affected individuals was 64.4 years [74]. When compared to the general population, the incidence appears to be much higher, suggesting that LS increases the risk of developing osteosarcoma. In addition, in spite of typically having a peak incidence during puberty, osteosarcoma in LS occurs at older ages [76]. Further evidence that osteosarcoma may occur in LS is provided by one retrospective study: sarcomas were found in 24 out of 178 LS patients. Among them, one female patient was diagnosed with osteosarcoma, as well as liposarcoma, in the absence of colorectal cancer. The patient had a family history of colorectal cancer and was diagnosed with LS and harbouring a *MSH2* (c.1661+1G>A) pathogenic variant. The authors were able to prove the pathogenicity of this variant (probable loss of heterozygosity was also present) [71].

Other cases of osteosarcoma in LS were presented by Lynch et al. [72] and Nilbert et al. [73]. The first authors identified a case of osteogenic sarcoma in a member of a family affected by LS underlying *MSH2* pathogenic variant (exon 4 splice site mutation TAG3 GAG, respectively), while the second group found a case of lower-leg osteosarcoma in a 15-year-old female carrying a *MLH1* (c.1276C>T) pathogenic variant [72,73]. Two further cases of bone cancer were cited in patients with *MSH6* pathogenic variant [75]. The link between LS and osteosarcoma might be explained by the involvement of the *MMR* genes, particularly *MSH2* [77,78,79]. In addition, a recent study linked a single nucleotide variant, rs17224367, located in the *MSH2* gene, to the prognosis of osteosarcoma in Chinese patients [80]. Another type of sarcoma reported both in LS and PDB was chondrosarcoma. Nilbert et al. [73] identified a case of chondrosarcoma in a 28-year-old male affected by a *MLH1* c.1204A>T, p.(Lys402Ter) pathogenic variant [73].

Considering that our patient has a pathogenic variant in the *MSH2* gene, it is crucial to consider the possibility of having a higher risk of osteosarcoma and even chondrosarcoma, both due to PDB and LS. While lifelong follow-up is required, further pathogenic studies will explain the connection between the conditions harbouring different genetic sets that, until now, has not been presented in the same patient.

### 3.7. Other Clinical Observations

As mentioned, our patient was identified with increased fasting plasma glucose and, further on, the diagnosis with type 2 diabetes mellitus was established. Additionally, she had high total serum cholesterol. One study from 2022 suggested that the components of metabolic syndrome are more likely to be found in patients with PDB than the general population [81]. Erol et al. [81] identified that nearly one third of the individuals with PDB might display different degrees of glucose profile anomalies [81]. Of note, our patient had normal blood pressure at the moment of mentioned endocrine assessments. The presence of diabetes might explain degraded bone microarchitecture as reflected by low TBS at central DXA, as well as the early menopause [82,83]. The effects of PDB on TBS are yet to be studied. On the other hand, in females with LS, early surgical menopause due to gynaecological cancers is prone to bone deterioration [84].

On the other hand, the patient had autoimmune Hashimoto thyroiditis with normal thyroid function. While thyroid cancer has been reported in LS, no particular association with this type of autoimmune endocrine condition is known [85,86]. We found one report of autoimmune thyroiditis in a subject with PDB [87]. Generally, this antibody-related disease has a higher prevalence in the general population, especially in individuals with other autoimmune diseases; however, its presence in this case might be incidental [88,89,90].

Concerning PBD, the presence of a single lesion as pointed out by bone imaging was very suggestive in addition to bone metabolism markers profile; thus, a bone biopsy was not considered as useful for differentiated diagnosis as it was deemed a “do-not-touch” lesion [91]. Furthermore, the genetic testing helped this distinction of PBD despite the fact that the patient had a relapse of the condition in terms of local pain and bone turnover markers. The most important differential diagnosis should be performed with a bone metastasis from her prior malignancy or other unidentified LS-associated cancers. However, at that point, no other cancer was active, and neither relapsed during follow-up [92,93,94,95].

Another particular aspect was the outcome of PBD in this adult female case. The relapse of the disease was associated with local pain, apparently non-responsive to mild analgesics for two episodes. The presence of increasing bone turnover markers was very suggestive in both cases, as pointed out in Figure 5. However, during the follow-up, the bone metabolism markers did not reach the high values from the first presentation. Currently, either symptomatic or intensive strategy is mainly focused on zoledronate use, which is effective in most cases as similarly seen in bone metastasis from different malignancies [17,32,96,97]. One seven-year study in PBD showed that a rate of 6% is associated with non-response to this therapy, while a rate of 15% was involved with a relapse after intravenous medication. Relapse prediction pointers were older age, higher ALP at baseline, and polyostotic PBD (which was not our case) [96]. Generally, zoledronate is considered the best option in PBD, also acting as anti-pain medication [17,97]. This rather complicated evolution was less expected in monostotic PDB, which covers less than one third of all cases. Forearm and fingers involvement in single-sited PDB have rarely been reported as well [98,99].

With regard to LS, this mentioned case belongs to the domain of real-life medicine, whereas practical guidelines are less or more applied. Of note, our (endocrine) follow-up took place amid the COVID-19 pandemic and some investigations were more difficult to be performed and/or accessed. Our patient had early onset colorectal cancer and gene testing was needed (ideally before surgery). The importance of identifying such pathogenic variants is reflected in deciding the type of surgical procedure such as colectomy or segmental resection. The presence of germline mutations indicates a higher risk than seen in later onset colorectal cancer and additional issues concerning fertility preservation and sexual health [100,101]. Moreover, in case of good evolution during surveillance, colonoscopy (and esophagogastroduodenoscopy in this case) may be repeated at 2–3 years in order to avoid unnecessary investigations [102,103]. However, the decision should be taken by a multidisciplinary team in this case as it is difficult to appreciate a more severe form due to the presence of the other mutation; hence, at the first one-year surveillance after the gene testing of both conditions, a more prudent approach was initially recommended.

In our case, iatrogenic (ovarian-related) hypogonadism was registered, the patient did not receive any hormone replacement therapy, and the endocrine check-up was performed only at the moment of PBD diagnosis. Oestrogens exposure after almost a decade since last menstruation is not recommended since usually a five-year therapeutic window should be taken into consideration. Of note, the patient had osteopenia with a high Z-score at central DXA, which was suggestive for a secondary cause of bone loss as found in early hypogonadism. At this point, the anti-resorptive medication that was necessary for PBD helps the bone mineral density as well. Gynaecological care for females diagnosed with LS also concerns the risk of endometrial cancer (the second most frequent cancer after colorectal malignancy in LS), as well as ovarian (and probably mammary), which is reported in 10% up to 90% of these subjects, depending on the study (including the importance of underlying mutation). In developed countries, endometrial and ovarian cancers represent the most frequent gynaecological malignancies, and 3% of each is caused by LS. The best long-term strategies rely on a multidisciplinary team or on excellent centres dedicated to such syndromes [104,105]. Whether the presence of PBD is prone to a particular phenotype of LS is still an open issue. 

## 4. Conclusions

This is the first case in which PDB co-occurred with LS. Both genetic defects that led to the two conditions appeared highly transmissible in the patient’s family, one from the mother and the other from the father. The patient might have an increased risk of osteosarcoma and chondrosarcoma, both due to PDB and LS. The phenotypic expression of the daughter remains uncertain so far, but long-term surveillance is needed. However, at this point, none of the pathological conditions from the mother or her family are detected. This female case report highlights the importance of genetic awareness starting from two distinct clinical presentations. Since this is a mostly unusual situation, we cannot emphasise the way one condition influences the outcome of the other, but lifelong surveillance is essential. Other particular aspects involve the presence of metabolic complications, which may be related to PBD and/or early iatrogenic hypogonadism due to prior malignancy as well as the relapse of the monostotic PBD that required a supplementation of the medical therapy. Finally, such an exceptional finding will hopefully provide a substantial degree of insight for future research, while other findings are yet to be identified during long-term follow-up of the proband and her relatives. 

## Figures and Tables

**Table 1:** Initial biochemical work-up of the patient with PBD and LS.

Parameter (Unit)	Value	Normal
AST (U/L)	22	5–34
ALT (U/L)	43	0–55
GGT (U/L)	40	6–42
Serum creatinine (mg/dL)	0.67	0.5–1.2
Serum uric acid (mg/dL)	4.6	2.5–5.7
Serum urea (mg/dL)	28.6	15–50
Total cholesterol (mg/dL)	254	0–200
LDL cholesterol (mg/dL)	189	60–160
HDL cholesterol (mg/dL)	42.7	40–65
Triglycerides (mg/dL)	119	0–149
Fasting glycaemia (mg/dL)	123	70–105
Glycated haemoglobin A1c (%)	6.5	<5.7
Total proteins (g/dL)	7.7	6.4–8.3
Serum sodium (mmol/L)	137	136–145
Serum potassium (mmol/L)	4.3	3.5–5.1
Serum magnesium (mg/dL)	1.9	1.6–2.6
PTH (pg/mL)	37.48	15–65
25-hydroxyvitamin D (ng/mL)	11.8	30–100
ALP (U/L)	347	40–150
P1NP (ng/mL)	203.4	20.25–76.31
ßCTX (ng/mL)	0.67	0.162–0.436
Serum total calcium (mg/dL)	10.3	8.4–10.2
Serum phosphorus (mg/dL)	3.2	2.3–4.7
24 h urinary calcium (g/24-h)	0.33	0.07–0.3

Abbreviations: AST = aspartate aminotransferase; ALT = alanine aminotransferase; ßCTX = type I collagen C-telopeptide; GGT = gamma glutamyltransferase; LDL = low-density lipoprotein; HDL = high-density lipoprotein; PTH = parathyroid hormone; ALP = alkaline phosphatase; P1NP = procollagen type 1 N-terminal propeptide (blue = abnormal levels).

**Table 2:** Dual-energy X-ray absorptiometry results (GE-Lunar Prodigy Pro device).

	BMD (g/cm^2^)	*t*-Score (SD)	Z-Score (SD)
Lumbar L1–L4	0.966	−1.7	−0.6
Total hip	0.979	−0.2	−0.2
Distal radius	0.67	−0.6	−0.6

Abbreviations: BMD = bone mineral density; SD = standard deviation.

**Table 3:** Denosumab use in patients diagnosed with PDB (the display starts from the oldest to the most recent report) [60,61,62,63,64,65].

First Author Reference Year of Publication	Study Type	Studied Population	PDB Type	Treatment	Biochemical and Scintigraphic Outcome	Clinical Outcome	Side Effects	Reason for Prescribing Denosumab	Other Observations
Schwarz [62] 2012	Case report	86-year-old male	polyostotic	denosumab 60 mg subcutaneously re-treated at months 6, 9, 12, and 15 with denosumab 60 mg	normalisation of plasma ALP and BALP; suppression of the other bone markers; less activity on bone scintigraphy (after 15 months of treatment)	improvement of pain	No	BP were CI due to impaired renal function	initially treated with calcitonin without effect
Verma [63] 2016	Case report	40-year-old male	polyostotic	6 doses of denosumab		15 months follow-up: asymptomatic and disease-free	No	3 years following treatment (pamidronate 60 mg every 3 weeks, calcium, and vitamin D supplements), the patient developed GCT	the patient also underwent surgery
Tanaka [64] 2016	Case report	60-year-old male	polyostotic	denosumab 120 mg on days 1, 8, and 15, and then once every 4 weeks		minimal symptoms	No	GCT	
Reid [65] 2016	Case report	75-year-old female	monostotic (skull)	denosumab 60 mg and a second dose after two years	ALP levels normalised for 4–6 months after each dose; less marked uptake than at baseline on bone scintigraphy	improvement of headaches and hearing	No	severe musculoskeletal pain after alendronate 40 mg orally	
Kostine [60] 2016	Case report	79-year-old male	polyostotic	Single-dose of denosumab 60 mg, oral calcium (1 g/day), and vitamin D (800 IU/day)	ALP levels normalised (ALP = 91 IU/L, BALP = 19 IU/L) within 4 months and remained low (BALP: 27 IU/L) after 18 months	rapid and marked pain relief	hypocalcaemia (a nadir of 1.1 mmol/L, ionised calcium: 0.54 mmol/L)	BP were CI due to impaired renal function	the patient did not take the prescribed calcium supplementation
Kuthiah [61] 2018	Case report	63-year-old female	polyostotic	denosumab 60 mg six-monthly	ALP levels normalised (118 U/L) within 3 months	pain resolved	No	BP were CI due to impaired renal function	

Abbreviations: ALP = alkaline phosphatase; BALP = bone alkaline phosphatase; BP = bisphosphonates; CI = contraindications; GCT = giant cell tumour.

**Table 4:** Summary of the findings regarding osteosarcomas and chondrosarcomas found in patients diagnosed with MMR pathogenic variants of germline type (LS); the first four references are case series of two cases per paper (*n* = 3) and one series of twelve individuals [71,73,74,75], and a single case report [72].

First author Reference Year	Population	Pathogenic Variant	Sarcoma Type	Other Tumours (Outside LS Panel)
Case series				
Carvalho [71] 2020	40-year-old female	*MSH2* c.1661+1G>A-LP	osteosarcoma	sebaceoma
	20-year-old female	*MSH2* c.2152C>T; p.Gln718Ter-P	osteosarcoma	liposarcoma
Dominguez-Valentin [74] 2020	5 males and 7 females with a mean age of 64.4	NA	osteosarcoma	-
Nilbert [73] 2009	15-year-old female	*MLH1* c.1276C>T; p.(Gln426Ter)	osteosarcoma	-
	28-year-old male	*MLH1* c.1204A>T; p.(Lys402Ter)	chondrosarcoma	-
Baglietto [75] 2009	2 patients	NA	bone cancer	-
Case reports				
Lynch [72] 2003	25-year-old male	*MSH2* exon 4 splice site mutation	osteosarcoma	-

Abbreviations: NA = not available; LS = Lynch syndrome.

## Data Availability

Not applicable.

## Abbreviations

ALP	alkaline phosphatase
BALP	bone alkaline phosphatase
CTX	type I collagen C-telopeptide
DXA	dual-energy X-ray absorptiometry
GCT	giant cell tumour
GWAS	Genome Wide Association Studies
LS	Lynch syndrome
MMR	mismatch repair
NTx	N-telopeptide
OPG	osteoprotegerin
RANKL	receptor activator of nuclear factor-kB ligand
P1NP	procollagen type 1 amino-terminal peptide
PCR	polymerase chain reaction
PDB	Paget’s disease of bone
PTH	parathormone (parathyroid hormone)
SQSTM1	sequestosome 1
TBS	trabecular bone score
99mTc-MDP	technetium-99m methylene diphosphonate
WES	whole exome sequencing
